# Strengthening district health systems to achieve Malawi’s decentralisation objectives: lessons learnt from Malawi’s Blantyre Prevention Strategy

**DOI:** 10.1136/bmjgh-2025-019811

**Published:** 2026-07-16

**Authors:** Betha O Igbinosun, Gift Kawalazira, Darwin Pangani, Sara M Allinder, Yohane Kamgwira, Deborah Hoege, Samden Seunda, Chimwemwe Mablekisi, Stephanie Heung, Jacqueline Crowell, Charles B Holmes, Beatrice Matanje

**Affiliations:** 1 Center for Innovation in Global Health, Georgetown University, Washington, District of Columbia, USA; 2 O’Neill Institute for National and Global Health Law, Georgetown University Law Center, Washington, District of Columbia, USA; 3 Blantyre District Health Office, Blantyre, Malawi; 4 Ministry of Local Government, Unity and Culture, Lilongwe, Malawi; 5 Malawi National AIDS Commission, Lilongwe, Malawi; 6 Department of Health and Social Services, Blantyre City Council, Blantyre, Malawi; 7 Clinton Health Access Initiative, Lilongwe, Malawi

**Keywords:** Global Health, Health policy, Health systems, HIV, AIDS

## Abstract

The Alma-Ata ‘Health for All’ Declaration of 1978 emphasised community participation in health systems and the need to ‘bring health care as close as possible to where people live and work’. Since then, decentralised approaches that leverage the proximity of local governments to their populations have emerged as the preferred model for health systems over fully centralised structures. The district health system, particularly in low- and middle-income countries, has evolved as a key framework to advance the Alma-Ata vision and strengthen health system governance in decentralised settings. However, implementation has often been hindered by limited local capacity, raising a persistent question: how can district health systems be strengthened to achieve decentralisation objectives? Using Malawi’s ongoing Blantyre Prevention Strategy (BPS), a systems-based project designed to build local capacity at district and city levels for HIV prevention, as a case study, this paper-reviews policies and frameworks guiding health sector decentralisation in Malawi and examines how BPS is contributing to strengthening the district health system and supporting broader decentralisation efforts in Malawi’s health sector.

**Summary box**

- Centralised governance structures in health systems often face challenges such as bureaucracy and inflexible top-down policies, prompting many low- and middle-income countries to adopt decentralised approaches for better responsiveness and equity.
- Despite its potential, decentralisation in health systems is often hindered by technical, administrative, financial and capacity constraints at the local level and verticalisation of disease-specific programmes by donors.
- Malawi’s Blantyre Prevention Strategy (BPS) exemplifies a district-based approach that advances health sector decentralisation objectives by strengthening local health system leadership while fostering community ownership and enhancing service delivery.
- BPS has bolstered Malawi’s HIV prevention response by taking a systems approach that has strengthened local capacity for effective targeting and surveillance; demand generation; quality, accessible service delivery; and sustained use of HIV prevention interventions.
- Elements of BPS are being adapted in other regions of Malawi, providing a scalable model for strengthening and integrating disease-specific programmes into subnational health systems while promoting sustainability, local ownership and intersectoral collaboration.

## Introduction

 Decentralisation is defined as ‘the transfer of authority, or dispersal of power, in public planning, management and decision-making from the national level to subnational levels, or more generally from higher to lower levels of government’.^1^ Decentralisation has been applied across diverse sectors and is underpinned by theories such as ‘close to ground’ and ‘watching the watchers’.^2^

The ‘close to ground’ theory emphasises that ‘effective and participatory governance requires that government structures be brought closer to the general population and that local institutions become channels through which people can both participate, contribute their resources for development, and express their needs to the central authority’.^3^ The ‘watching the watchers’ theory speaks to the accountability and oversight between levels of government and is typified by regulatory provisions emphasising hierarchical structures and conferring oversight responsibility to central governments over local government actors.^2^

In the health sector, decentralisation reforms were bolstered by the ‘Declaration of Alma-Ata’ in 1978, which espoused a ‘Health for All by the Year 2000’ objective to be achieved by community participation and making healthcare universally accessible by bringing healthcare services ‘as close as possible to where people live and work’.^4^ The Declaration of the WHO-organised Harare Conference on Strengthening District Health Systems Based on Primary Health Care (the ‘Harare Declaration’) in 1987, endorsed district health systems as the means to achieve the Alma-Ata objective.^5^ It defined a ‘district’ as ‘a unit of the national health system which at the same time enjoys a certain degree of autonomy with respect to establishing health priorities, based on local needs, [operating] within a clearly defined geographical and administrative unit of local government’.^6^

In the decades following the Harare Declaration, many sub-Saharan African countries decentralised their health systems to complement the democratisation of their governance structures.^7^ These health systems embraced the district health strategy, organising and coordinating the delivery of health services at local levels and implementing a tiered system for the organisation of health facilities.^8^ Outside the region, countries such as Spain, Brazil, the Philippines, Colombia, Portugal, Indonesia and Pakistan have also implemented health-sector decentralisation reforms for reasons including fiscal imbalances; limited coverage, accessibility and quality of services; inequities in access, particularly in rural and underserved areas; weak coordination; and poor health outcomes.^9 10^

While centralisation offers benefits such as standardising policies, streamlining programmes and reducing duplication, it can limit responsiveness to local needs.^11^ Such responsiveness is particularly vital in the health sector, where tailoring services to community contexts ensures their suitability and acceptability. Decentralised approaches, by conferring greater decision-making authority on local authorities who better understand community needs, help ensure that priorities and interventions are appropriate to local contexts.^12^ Indeed, ‘healthcare solutions need to be context-specific to be truly effective’^13^ and ‘bottom-up’, decentralised systems foster this by bringing health services closer to populations in need, promoting accountability and equity within the health system, enhancing local control and strengthening community participation in planning and administration.^14 15^

However, the effectiveness of decentralisation reforms has been hindered by capacity constraints within local authorities, limited fiscal resources at local levels, ambiguous roles and responsibilities, lack of accountability in resource use and political interference.^16^,^18^ Effective decentralisation requires adequate institutional capacity, meaning that institutions with decentralised authority can ‘anticipate and influence change, make informed decisions, attract and absorb resources and manage resources to achieve objectives’.^19^ Simply ceding powers to local authorities is insufficient unless they have the necessary technical, administrative, financial and organisational capacities to perform their functions.^20^

To achieve its decentralisation objectives, Malawi has prioritised strengthening district-level governance structures. Following its transition from a highly centralised governance system in 1994, the country implemented decentralisation reforms across multiple sectors, devolving management responsibilities to district and city councils.^17^ In the health sector, the Ministry of Health devolved responsibility for primary and secondary-level health services to district health offices, and efforts are ongoing to develop operational guidelines to assist district councils in managing the decentralised district health system.^21^ Congruently, Malawi’s latest Health Sector Strategic Plan III (2023–2030) (HSSP III) outlines strategies to accelerate progress toward achieving Universal Health Coverage targets by 2030, including ‘decentralizing service delivery and empowering communities to take full ownership of the system at service delivery level’.^22^

Drawing on the Harare Declaration Action Plans for Strengthening District Health Systems, as well as programme documentation, peer-reviewed publications, routine activity reports, implementation experience and external evaluation findings, this practice paper examines how the Blantyre Prevention Strategy (BPS) supported the institutional capacity of Blantyre City and District Health Offices to implement core HIV prevention functions within Malawi and Blantyre’s broader national and district HIV response context. These functions include identifying populations at higher risk of HIV infection, generating demand for HIV prevention services, delivering quality prevention services and enabling the effective and sustained use of prevention services. The paper provides an overview of Malawi’s decentralisation framework and its implementation in the health sector, and analyses six key domains through which BPS is strengthening decentralisation efforts in Malawi’s health system by embedding core public health functions within the district health system. Our objective is to generate practice-based learning on how district-level health system capacity can be strengthened in a decentralised setting.

## Malawi’s administrative structure

Malawi is a landlocked country in southeastern Africa, with an estimated population of 21.1 million people in 2023.^23^ After its independence in 1964, Malawi maintained a highly centralised, single-party government until 1994, when it transitioned to a multiparty democracy. The newly elected government attributed the limited development progress and persistent poverty to the absence of effective local governance structures. Consequently, it initiated reforms to decentralise administrative and political authority as a mechanism for realising its development strategy.^24 25^

**Figure 1:**
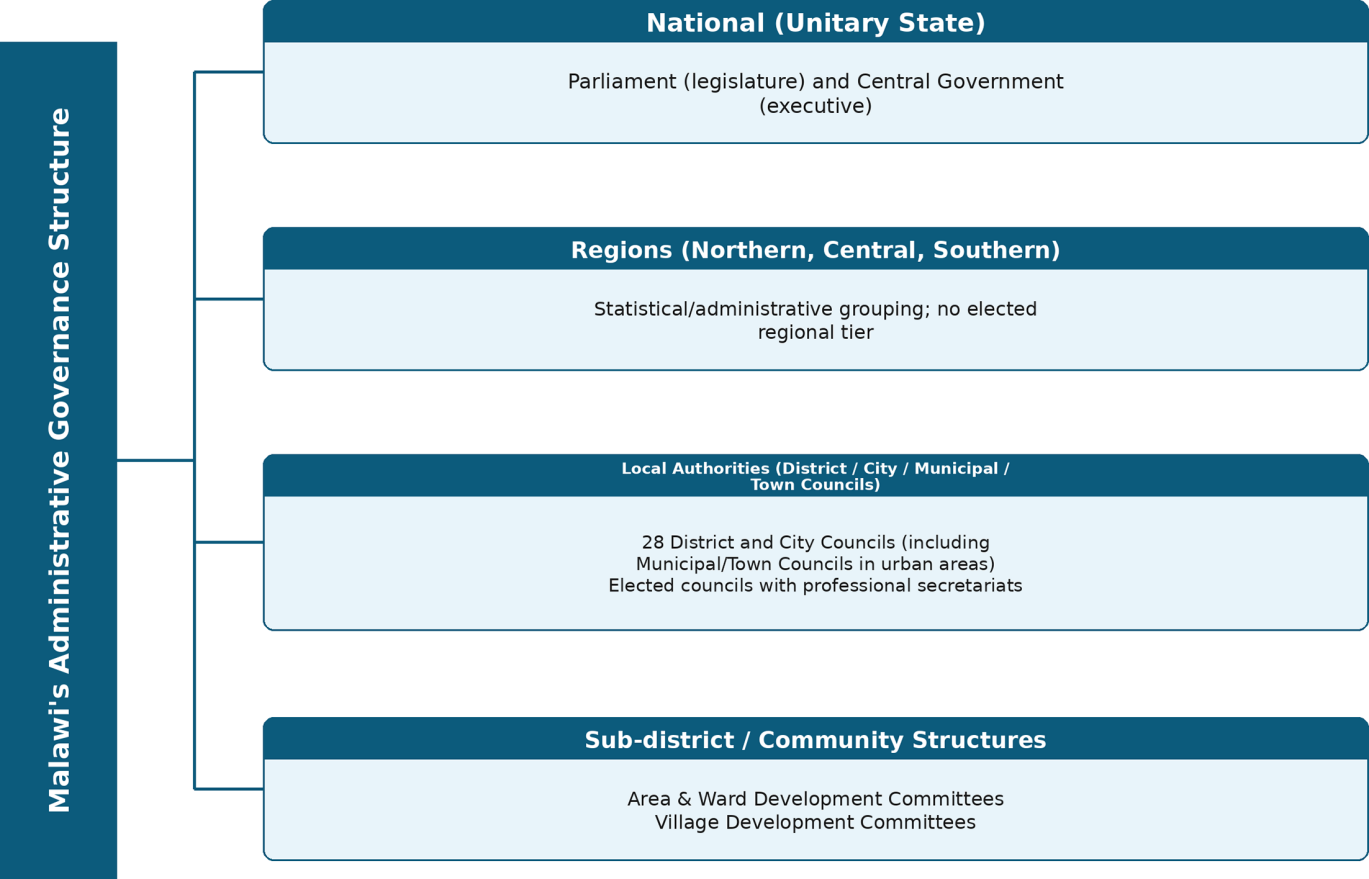
Malawi’s administrative structure.

In 1995, Malawi adopted a new constitution that established local government authorities as a key part of the country’s democratic system and granted parliament the authority to delegate local policy and administration matters to these authorities.^26^ In 1998, the government passed the Local Government Act, empowering these authorities to administer local government areas, make local governance and development policy decisions, mobilise resources and create by-laws for the local government area, among other responsibilities.^27^ The National Decentralisation Policy, also enacted in 1998, operationalised the decentralised structure by codifying administrative and political authority devolvement to districts.^28^ Malawi operates a single-tier decentralised system comprising 28 district councils, 4 city councils, 2 municipal councils and 1 town council; the three regions (Northern, Central, Southern) are used for geographical/statistical coordination and do not constitute a devolved governance tier.^29^ Figure 1 illustrates Malawi’s administrative structure.

## Decentralisation in Malawi’s health sector

Following adoption of the National Decentralisation Policy in 1998, the Ministry of Health in 2005 issued Guidelines for the Management of Devolved Health Service Delivery by District Councils, which conferred managerial autonomy over health service delivery to district councils.^30^ In 2015, as part of the government-wide Public Sector Reform Programme, the Ministry of Health initiated the process of fully decentralising the district health system. This process is ongoing, with next steps focused on developing District Health System Operational Guidelines to assist District Councils in managing the decentralised district health system and improving coverage, efficiency and quality of services in line with the Health Sector Strategic Plan and the Sustainable Development Goals.^21^

Currently, districts oversee primary and secondary-level health services while the central Ministry of Health retains responsibility for overall stewardship of the health sector, including policy development, standard setting, quality assurance, supervision and technical support, as well as oversight of tertiary-level health services.^30 31^ The National AIDS Commission also provides overall leadership and coordination to ensure a multisectoral national response to the HIV and AIDS epidemic.

At the zonal level, five Zonal Health Support Offices—Northern, Central East, Central West, South East and South West—support national coordination and provide technical assistance to districts in planning, supervision and monitoring health services.^31^ At the district level, the District Health Office (DHO) coordinates, plans, supervises and monitors implementation of the district health agenda. Each DHO is led by a Director of Health and Sanitary Services (DHSS), supported by senior officers including the District Medical Officer, District Nursing Officer, District Environmental Health Officer, District Health Promotion Officer and District Health Services Administrator. The District Health Management Team (DHMT) includes these principal officers and serves as the DHO’s decision-making body responsible for planning, organising, monitoring and evaluating district health services.

Each district council has a Principal Nutrition, HIV and AIDS Officer, who coordinates and supports the implementation of nutrition and HIV/AIDS programmes within the district. At the facility and community levels, Health Centre Management Committees (HCMCs), Health Surveillance Assistants and Village Health Committees facilitate service delivery, community engagement and health promotion.^31^

Within Blantyre City, the Directorate of Health at the City Council is led by the City’s Director of Health and Social Services, supported by a deputy and an assistant director. These officers, along with the Senior Environmental Officer and eight section heads, including those for HIV and AIDS and social welfare—jointly constitute the Health Management Team.

**Figure 2:**
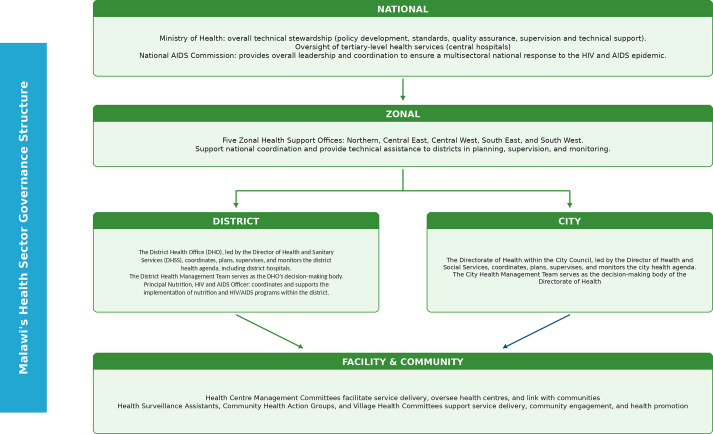
Malawi’s health sector governance structure.

Decentralisation has improved Malawi’s health sector by enabling locally tailored health programmes and enhancing community accountability.^32^ However, challenges persist, including limited autonomy of health facilities, lack of community engagement, inadequate fiscal resources, weak coordination, insufficient capacity among programme coordinators and other personnel for effective health planning and management and limited accountability.^32 33^ As a result, Malawi has struggled to make progress in some key health areas. For example, while Malawi has made significant progress in its HIV response, more than 900 000 people lived with HIV and almost 12 000 new infections were recorded in 2024.^34^ Key populations with the highest HIV burden include female sex workers and people who inject drugs. The epidemic remained concentrated in the southern region, which accounted for more than 57% of new infections in 2024.^34^ Figure 2 illustrates Malawi’s Health Sector Governance Structure.

## Overview of Malawi’s Health Sector Strategic Plan III (2023-2030)

Following the lapse of Malawi’s Health Sector Strategic Plan II (2017–2022), the HSSP III (2023–2030) was drafted through a consultative process overseen by the Health Sector Working Group and managed by the Ministry of Health’s Department of Planning and Policy Development, which included district teams.^22 35^ This process was part of efforts to move Malawi from reliance on external funding towards universal health coverage and to ensure the delivery of cost-effective, high-quality, accessible, equitable, affordable and sustainable healthcare services.^22^

The HSSP III recognises that centralised control of the health sector restricts district-level strategic thinking, accountability and priority setting.^35^ Accordingly, the HSSP III outlines requirements such as joint planning, prioritisation, implementation and monitoring of all health system activities within a ‘One Plan, One Budget, One M&E’ framework. It requires (1) creation at district level of a unified list of priority health activities, drawn from across the health system, including health centres and district hospitals; (2) decentralised funds management by district councils and central hospital boards and direct facility financing; (3) decentralised, interoperable health information systems at community, facility and district levels; and (4) strengthened community oversight of local healthcare delivery.^22^

## Vital domains for strengthening district health systems

The pertinent question, then, is what is required to enhance decentralisation efforts in health systems? In other words, what functional capacitation is necessary to effectively strengthen district health systems? Blantyre District in Malawi’s southern region offers answers to these questions and how to operationalise those functions.

### Blantyre context

Prior to BPS, Blantyre was the epicentre of Malawi’s HIV epidemic.^36 37^ Blantyre has a population of about 1.5 million people, nearly 48% of whom are under 15 years of age.^37^ In 2016, Blantyre City’s adult HIV prevalence was 17.7%, the highest among Malawi’s 28 districts and almost two times the national average of 10%.^36 37^ Several factors contributed to this, including Blantyre’s status as the country’s oldest urban centre and commercial capital, which attracts many young people and migrant workers seeking economic opportunities.^38^

While Blantyre has been a priority district for implementing HIV programmes in Malawi, many activities were implemented in a fragmented manner by numerous local faith-based, non-governmental and community-based organisations (NGO/CBO), as well as international partner affiliates.^38^ The district also lacked the systems and resources needed to coordinate these diverse actors and interventions, resulting in activities that were often poorly aligned with community needs.^38^

### Blantyre prevention strategy

**Figure 3:**
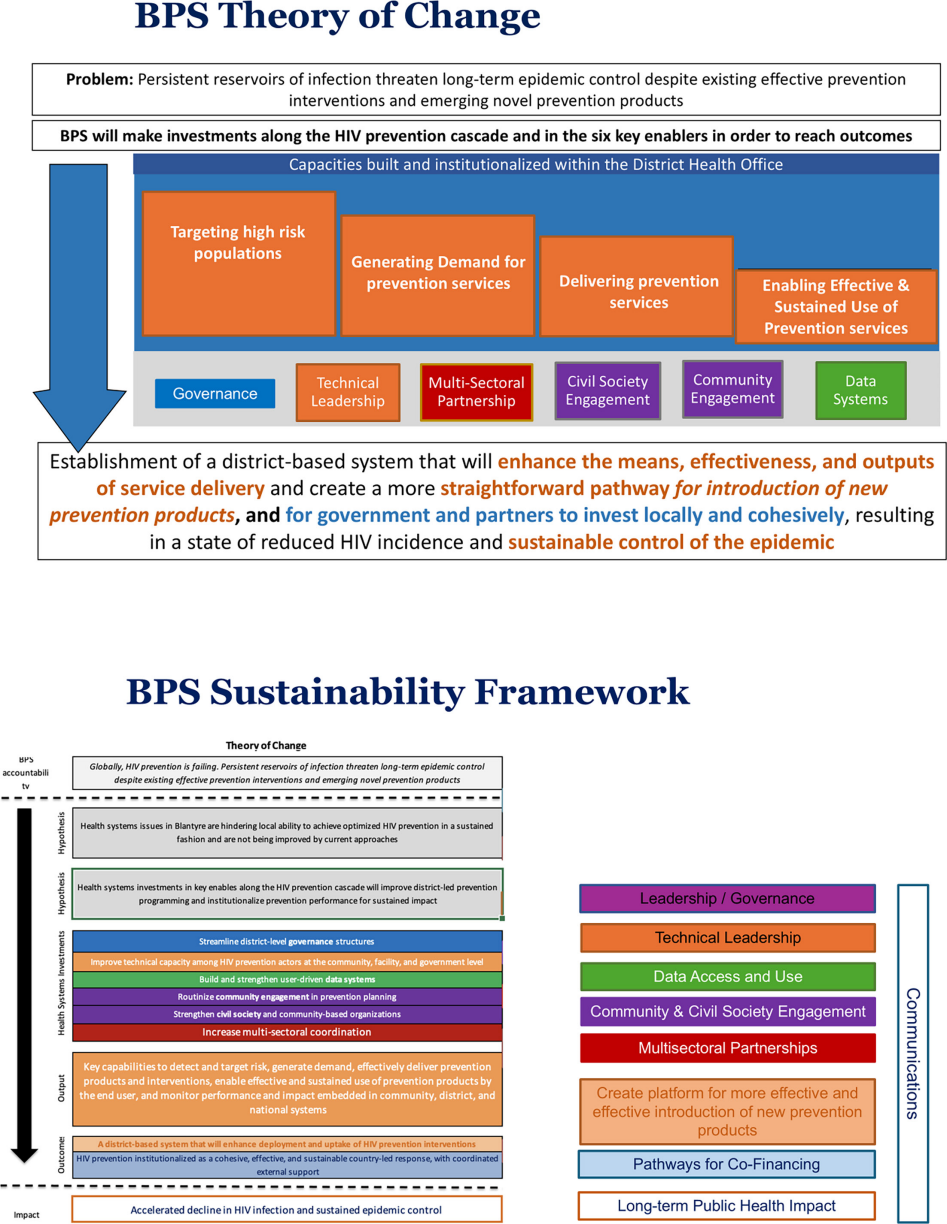
BPS theory of change. BPS, Blantyre Prevention Strategy.

In response to these challenges, BPS was launched in May 2020, under the leadership of the Government of Malawi, with support from the Center for Innovation in Global Health at Georgetown University and other partners, and funding from the Bill and Melinda Gates Foundation. It was designed as a counterweight to vertical or parallel programmes that may have some public health benefit but further fragment subnational health systems. The project aimed to develop a robust local system for sustained HIV prevention and institutionalise a cohesive, effective and sustainable country-led HIV prevention response with coordinated external support. To test the hypotheses within the BPS theory of change, its holistic approach channelled investments towards strengthening and embedding essential functions and capabilities within existing district-level structures for effective targeting and surveillance, demand generation, service delivery and sustained use of HIV prevention interventions. (See figure 3: BPS Theory of Change).

Over the course of 3 years, national and local government staff, BPS-supported partners and others adapted previously tested approaches to Blantyre, co-developed plans, trained local staff and implemented activities. BPS applied adaptive learning techniques to course correct and try new approaches based on data and experience. In year 4 (May 2023 to April 2024), leadership of activity planning and implementation shifted from being largely BPS-funded partner-driven to ownership by the District and City Health Teams.^39^

The effects of BPS have been far-reaching,^37^ enhancing ‘district leadership capacity’ to coordinate previously fragmented multisectoral partners working in Blantyre and strengthening district health system capacity from communities up to elected officials.^40^ BPS has also supported quality improvement initiatives that increased pre-exposure prophylaxis (PrEP) uptake in Blantyre; use of data and insights from ‘community labs’ (structured community engagement sessions using simplified human-centred design methods) to inform service delivery priorities; and implementation of HIV surveillance, detection and response activities.^40 41^

Coinciding with BPS implementation, and within the context of Malawi’s broader national and district HIV response, Blantyre’s HIV prevalence declined from over 17% in 2016 to 10% by 2025 and the district is no longer the one with the highest HIV prevalence in Malawi.^37^ While these trends cannot be attributed solely to BPS, an external evaluation of BPS found that ‘BPS significantly increased HIV testing, sustaining an additional 533 tests per month. Similarly, …PrEP uptake, screening and initiation improved, with PrEP initiation rates rising from 19.8% in early 2021 to 65.1% in early 2024’.^40^

With BPS project implementation in Blantyre scheduled to conclude on 31 July 2026, the focus in its final year is on documenting and institutionalising the systems components needed for HIV prevention within the Blantyre District and City public health system. Among these components are the functions, capabilities and relationships to ensure the district and city health teams continue to lead the coordination of HIV services across facilities, partners and communities; deliver services that are high-quality, data-driven, people-centred and non-stigmatising; detect and respond rapidly to HIV risks; and co-design health promotion and prevention interventions with communities.

The section below maps the various domains of the Harare Declaration Action Points against BPS activities to describe how BPS is strengthening Blantyre’s District Health System.

### Mapping Blantyre Prevention Strategy-supported strengthening of Blantyre’s District Health System to the Harare Action Points

**Figure 4:**
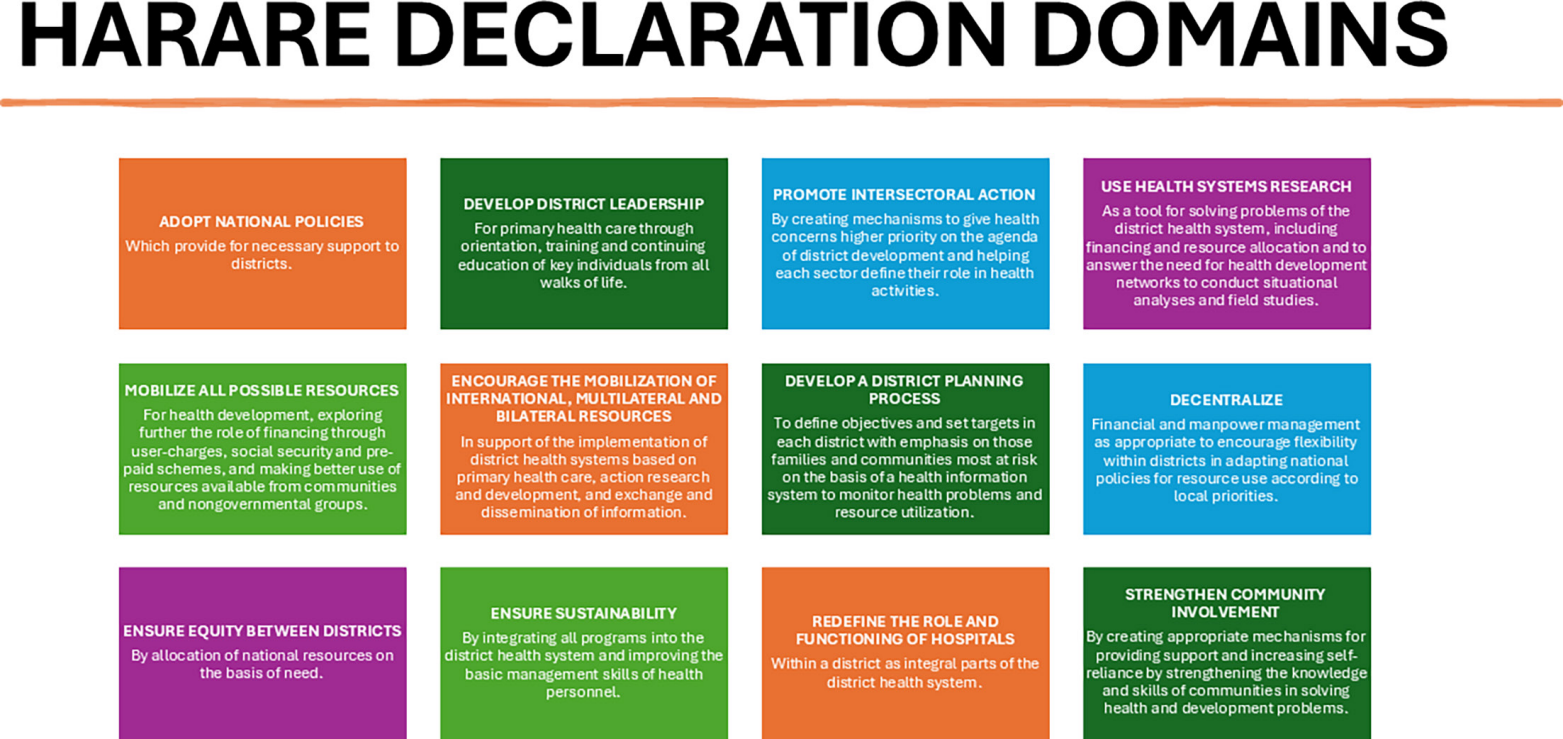
Declaration of the Harare Conference on Strengthening District Health Systems (Action Points).

The Declaration of the Harare Conference identified several action points for strengthening district health systems (see figure 4).^5^ This section draws on evidence from BPS programme documentation, peer-reviewed publications, routine activity reports, implementation experience and external evaluation findings.^40^ Using these sources, BPS activities were mapped against the Harare Declaration Action Points to examine how the project has strengthened the district health system in Blantyre and built institutional capacity to enhance the effectiveness of decentralisation efforts in Malawi’s health sector. Together, these domains show how BPS supported Malawi’s decentralisation objectives by strengthening district capacity to plan and prioritise using local data, deliver quality services, mobilise resources, engage communities, coordinate stakeholders and promote intersectoral action.

#### Supporting district planning

The objective of health systems planning is to enhance the delivery of health services and improve population health outcomes.^42^ Achieving this goal requires district health systems to be able to (1) develop plans for health services, (2) determine district priorities (which may differ from national priorities), (3) involve stakeholders in the planning process and promote alignment of stakeholder activities and funding with district priorities and (4) implement district plans.^43 44^ Integral to this process is a robust health information system that facilitates effective planning and decision-making.

BPS supports district planning by improving routine access to and use of data for informed HIV and health-related decision-making. BPS invested in creating a data pipeline, linked to national systems, that synthesises multisectoral data sources into the Prevention Adaptive Learning and Management System (PALMS), a user-friendly digital dashboard. PALMS was developed from Blantyre district-based health data users’ insights about their needs and preferences for data presentation and use. PALMS presents data from the pipeline that allows DHMT coordinators to monitor relevant disease and programme performance-related indicators across HIV and other health programmes while also enabling data access for decision-making by site/community-level individuals. Having necessary data in a single, easy-to-access digital health platform has improved access to data at district, facility and community levels, along with data use and data-informed planning and decision-making.

In addition, BPS built capacity for passive and active HIV surveillance within the DHO’s existing Integrated Disease Surveillance and Response (IDSR) unit, district coordinators and facilities through pilot use of IDSR approaches for HIV prevention. District, facility and community health workers can now identify upticks in new infections and/or proxy risk for infection by geographical area and subpopulation, target services to those communities or populations and coordinate and monitor the performance of government staff as well as NGO/CBO partners operating in the district.

To support institutionalisation and sustainability, the Ministry of Health Digital Health Division (DHD) assumed management of PALMS and the data pipeline in 2025. Blantyre’s new data-driven, decision-making culture is on its way to meeting HSSP III objectives, which include further decentralisation of the health information system to each decision-making entity at every level of the health system, aiming to foster ‘fully decentralized yet optimally integrated, fit for purpose, [Health Management Information Systems] at the community, health facility, district, [and] central hospital [.]’.^22^

#### Strengthening district leadership

District leadership is crucial for ensuring effective programme implementation and quality service delivery.^43 44^ BPS has worked to enhance the district health team’s governance and technical leadership skills in all aspects of the HIV prevention system, creating connections between the district and city health teams and between other systems actors, which has improved coordination and created a more seamless, whole-of-district HIV response.^40^ One focus area is strengthening the leadership and operational capacity of the DHO’s quality improvement (QI) unit. Extensive training and mentorship were provided to the district Quality Manager, QI ‘mentors’ (District Coordinators) and focal persons at district and facility levels to apply QI methodologies and develop, test and implement change ideas for QI projects. Quality Improvement Support Teams at both levels have become the centre of the district’s data review and response activities. The DHO has used these systems investments and tools to address other health challenges, including a cholera outbreak and respond to other emerging public health needs caused by Cyclone Freddy in 2023.

#### Bolstering community involvement

Community involvement is strengthened by ‘creating appropriate mechanisms for providing support and increasing self-reliance by strengthening the knowledge and skills of communities in solving health and development problems’.^5^ Active community involvement in planning health services can promote service responsiveness and ensure that health interventions reflect local needs.^45 46^ Under DHSS leadership, BPS instituted a systematic approach that enables district staff and partners to elicit community insights from clients and community members about HIV service delivery challenges and opportunities for improvement. It adapted a ‘community lab’ model to the Malawian context in which trained staff use simplified human-centred design methodologies to generate insights that the district and partners can use to address identified issues. The laboratories have fostered feedback loops between communities, health facilities and the district and city health offices that enable people-centred programming. For example, insights gathered from community labs identified barriers to PrEP delivery due to the stigma around its delivery at HIV treatment clinics within facilities. This information was incorporated into a district-led QI collaborative, and a change idea was tested, which increased access to PrEP delivery at additional clinics within the facility. In addition, the information informed changes to the national PrEP guidelines.

Based on early project implementation, in the project’s third year (May 2022 to April 2023) the district established linked networks between communities, facilities, the district, the city and others to align the work of CBOs with health facilities and other partners. Network committees centred around public health facilities have become the platform for multisectoral partners to jointly review data and coordinate activities to address local needs within the catchment area. Facilitated by district coordinators, these committees seek to strengthen the coordination of fragmented multisectoral delivery and community channels, promote shared resource allocation and improve service delivery and health outcomes. Importantly, the networks enable community leaders to play an active role in decision-making processes for community activities, fostering community empowerment objectives espoused in the Harare Declaration and HSSP III.

#### Promoting intersectoral action

Intersectoral action is shaped by the broad conceptualisation of health beyond a biomedical approach, encompassing social, economic, political and environmental factors that influence population health.^47 48^ Promoting intersectoral action requires ‘creating mechanisms to give health concerns higher priority on the agenda of district development and helping each sector define their role in health activities’.^5^

Under BPS, efforts were made to engage local government leaders in Blantyre who had become disengaged from the HIV response over time as ‘others’—largely externally financed international NGOs—had taken on more of the response. A structural risk reduction working group, consisting of local political leaders (Ward Councillors) from the City Council, was established to re-engage them in addressing the multifaceted social and structural drivers of HIV in Blantyre, including poverty, unemployment, alcohol and substance abuse and transactional sex. It was later expanded to include District Councillors.^49^

BPS co-developed a training programme that increased councillor capabilities in data access and interpretation, advocacy, community engagement and resource mobilisation. Following the trainings, the Councillors launched HIV prevention efforts in their respective wards, for example, integrating HIV prevention messages into their routine activities, visiting health facilities and engaging with implementing partners to ensure accountability.^50^ It is anticipated that Councillors will use their BPS-supported capacitation to act at council level, including enforcing existing or enacting new by-laws to address structural risks, further promoting intersectoral action.

#### Mobilising resources

Resource mobilisation is vital for effective health system strengthening. Domestic resources are scarcely sufficient to finance health services in low- and middle-income countries, but external assistance can often be vertical, targeting specific services.^51^ While donors recognise the benefits of channelling funds through local institutions and understand how the prevalence of vertical disease programmes can lead to health system fragmentation, lack of accountability and limited capacity lead them to bypass state institutions.^52^,^54^ Overcoming such fragmentation and promoting country ownership of health plans and priorities requires creating attractive opportunities for donor investment, for example, local institutions with demonstrated capacity to use such funding effectively.^52^

By investing in strengthening district-level capacity to execute vital health system functions, BPS creates opportunities for further domestic and external investment. As evidence, Blantyre district collaborated with health facilities and implementing partners to incorporate activities that enable operationalisation of the BPS-supported health system functions into the 2024 District Implementation Plan, which set out its funding priorities. This strategic inclusion supports the sustainability of these functions, promotes a less fragmented funding environment and creates a platform to mobilise resources for broader health system strengthening.

Additionally, Blantyre is one of the pilot districts selected for direct facility financing (DFF), with 12 facilities participating in the pilot. The selection criteria included the location of the facility (rural vs urban), the number of clients served and, most importantly, the functionality of the HCMC. Training of HCMCs supported by BPS helped activate some of these committees, thereby qualifying their facilities for DFF. Facility staff and HCMCs have been trained in planning, budgeting and financial management. As part of sustainability efforts, facilities will incorporate risk identification and response mechanisms within DFF processes and will receive support for mobility through budgeted items such as fuel, data bundles and other allowable expenses under DFF guidelines, while programme coordinators will budget for capacity-building initiatives for the participating facilities.

#### Ensuring sustainability

Sustainability is crucial to protecting investments in public health systems and maximising their public health impacts.^55^ Vertical programmes, which are often rigid, centrally planned, narrowly targeted and managed separately from routine health services, limit sustainability within the local system.^14 18 56 57^ The absence of integration and inability of district health managers to administer these programmes deters sustainability and broader health system strengthening.^18^ Moreover, unsustainable activities or projects result in the waste of financial, technical and human investments, low levels of community support and reduced trust in health systems.^55^ They also create vulnerabilities in the health system when funding disruptions occur, as experienced in 2025.^58^

Ensuring sustainability requires ‘integrating all programs into the district health system and improving the basic management skills of health personnel’.^5^ BPS’s objective is not to sustain specific activities but rather to institutionalise the capabilities and relationships needed for effective HIV prevention within the health system, ensuring they become routinised across all of Blantyre’s HIV prevention activities and other disease categories as part of a broader integrated health response.

Affordability importantly contributes to sustainability. BPS has endeavoured to create a model that is affordable for sustainability and adaptable to other contexts with minimal additional cost. For example, BPS built the data pipeline on existing interoperable digital health platforms and transferred PALMS ownership to the DHD. BPS collaborated with national mobile network operators in Malawi to enable reversible billing for users with airtime limitations and developed a mobile application that allows offline access to archived data when the internet is unavailable. These efforts contribute to the sustainable integration of BPS-supported tools and functions into the health system for affordable, broader use.

## Policy lessons for district-strengthening interventions

While BPS was launched in Blantyre, several of its core elements, including data access and use, community insights and QI, were adapted in Lilongwe District to support the introduction of long-acting injectable PrEP through Malawi’s PathToScale Injectable PrEP Initiative, launched in September 2023. In April 2025, the Malawi National AIDS Commission launched the Strengthening District Health Systems for Sustainable HIV Prevention Working Group, comprising government, donor and partner organisations, to develop strategies for strengthening district public health systems across Malawi based on the BPS ‘model for developing subnational capabilities for more effective and locally led HIV prevention service delivery’.^39^ Implementation planning is underway in three initial high HIV-incidence districts.

Further institutionalisation and sustainability efforts include embedding BPS programme elements within District Implementation Plans and translating BPS learnings into national policies and guidelines. A multimedia BPS toolkit is under development to guide future adaptation in Malawi and elsewhere. Together, these actions are designed to embed the BPS model within Malawi’s health system and position it as a foundation for future programme implementation within and beyond the country.

Implementation has faced several challenges, including the redeployment of BPS-trained staff outside the district, funding reductions following the US Government Stop Work Order that led to the termination of partner agreements, and limited capacity within the DHD to assume full handover of PALMS. Despite these constraints, BPS has strengthened the HIV response in Blantyre and offers a practical model for building local institutional capacity.

Although specific approaches will vary by local and national context, investing in the BPS model can strengthen health system capacity, promote country ownership and ensure long-term sustainability, particularly amid declining development assistance for health. Transferability depends on strong health system governance and national and district-level commitment to subnational capacity-building. While no BPS element is wholly specific to Blantyre, some elements, such as data use for targeting and district-led quality improvement, may be more readily adapted to other districts, countries and disease areas because they align with broader public health functions and can be embedded within existing planning, monitoring and service delivery processes.^59^ Other elements may require greater contextualisation. For example, community laboratories depend on the existence and acceptability of direct feedback loops between communities, facilities and government actors, while engagement of elected officials on social and structural drivers of HIV risk may depend on local governance arrangements, political priorities and willingness to engage with social determinants of health.

Beyond HIV prevention, BPS-supported functions may be relevant to other public health priorities that require data-informed targeting, quality service delivery, systematic community engagement, sustained service use and coordinated action across facility and community settings. These functions may be particularly relevant for non-communicable diseases, where prevention and long-term management require continuous engagement with individuals and communities. They may also inform district-level approaches to health security priorities, and Blantyre’s use of BPS-strengthened systems for cholera response and emerging needs following Cyclone Freddy illustrates the potential relevance of these capacities beyond HIV prevention.

As international funders implement sustainability strategies, investing in district-level functionality can help fulfil their goals to strengthen the institutional, technical and service delivery capacity of the public health system in partner countries.^60^ Strengthening district governance and coordination across regionally dispersed services is also critical to sustaining decentralised efforts and improving service delivery across the health system. Recommended actions include establishing regional coordination mechanisms that convene clusters of districts for joint planning and data review, adopting shared performance indicators and interoperable dashboards and defining partner roles through memoranda of understanding and coordinating frameworks aligned with district plans and budgets.

### Limitations

This practice paper draws on programme experience and routine documentation rather than a formal research design and does not attempt to attribute outcomes solely to BPS. The insights presented are descriptive and largely based on implementation in Blantyre and would require adaptation for other districts or settings.

## Conclusion

Health sector decentralisation efforts recognise that the varied nature of population health needs requires a localised and flexible approach, which ensures that the health services provided address the needs of local populations and are accessible to all. However, the critical question remains: How can the capacity of local health systems be strengthened to address these needs? Rather than narrowly focusing on a single HIV prevention intervention, the BPS model stands out for its emphasis on building capacities within existing Blantyre City and District structures. Its approach has ensured that the developed capacities are integrated into the local health system, strengthening decentralisation and fostering sustainability.

## Data Availability

Data sharing not applicable as no datasets generated and/or analysed for this study.

## References

[1] Mills A, Vaughan JP, Smith DL (1990). Health system decentralization: concepts, issues and country experience.

[2] Abimbola S, Baatiema L, Bigdeli M (2019). The impacts of decentralization on health system equity, efficiency and resilience: a realist synthesis of the evidence. Health Policy Plan.

[3] SADC (Southern African Development Community) and UNDP 1998 SADC regional human development report.

[4] World Health Organization (1978). Report of the International Conference on Primary Health Care Alma Ata, USSR, 6–12 September 1978.

[5] (1987). Declaration of the Harare Conference on Strengthening District Health Systems Based on Primary Health Care. https://iris.who.int/handle/10665/61829.

[6] (1987). Strengthening district health systems, Harare, 3-7 August 1987. https://www.ircwash.org/sites/default/files/71WHO87-3827-strength.pdf.

[7] Eboreime EA, Nxumalo N, Ramaswamy R (2018). Strengthening decentralized primary healthcare planning in Nigeria using a quality improvement model: how contexts and actors affect implementation. Health Policy Plan.

[8] Meessen B, Malanda B (2014). Community of Practice “Health Service Delivery”. No universal health coverage without strong local health systems. Bull World Health Organ.

[9] Couttolenc BF (2012). Decentralization and governance in the Ghana health sector. https://openknowledge.worldbank.org/bitstreams/a529821b-6fe0-597a-b9a3-66d29b1f4bce/download.

[10] Mahmood S, Sequeira R, Siddiqui MMU (2024). Decentralization of the health system – experiences from Pakistan, Portugal and Brazil. *Health Res Policy Sys*.

[11] Krenyacz E, Revesz EE (2025). Consequences of centralized healthcare systems: changing role and autonomy of hospital managers - insights from a Hungarian case. J Health Organ Manag.

[12] Sreeramareddy CT, Sathyanarayana TN (2013). Decentralised versus centralised governance of health services. Cochrane Database Syst Rev.

[13] Dumka N, Gurung A, Hannah E (2024). Understanding key factors for strengthening Nepal’s healthcare needs: health systems perspectives. J Glob Health Rep.

[14] Henriksson DK, Ayebare F, Waiswa P (2017). Enablers and barriers to evidence based planning in the district health system in Uganda; perceptions of district health managers. BMC Health Serv Res.

[15] Mody J (2004). Achieving accountability through decentralization: lessons for integrated river basin management. World Bank policy research working paper no. 3346. https://hdl.handle.net/10986/14045.

[16] Gilson L, Kilima P, Tanner M (1994). Local government decentralization and the health sector in Tanzania. Public Admin Dev.

[17] Jagero N, Kwandayi HH, Longwe A (2014). Challenges of decentralization in Malawi. International Journal of Management Sciences.

[18] Heerdegen ACS, Gerold J, Amon S (2020). How Does District Health Management Emerge Within a Complex Health System? Insights for Capacity Strengthening in Ghana. Front Public Health.

[19] Rondinelli DA, McCullough JS, Johnson RW (1989). Analysing Decentralization Policies in Developing Countries: a Political‐Economy Framework. Dev Change.

[20] Bossert TJ, Mitchell AD (2011). Health sector decentralization and local decision-making: Decision space, institutional capacities and accountability in Pakistan. Soc Sci Med.

[21] Ministry of Health Malawi (2018). Report on the assessment on the current state of district health system decentralisation carried out from 21st to 31st May, 2018. https://p4h.world/en/documents/assessment-of-the-current-state-of-district-health-system-decentralization.

[22] Government of the Republic of Malawi (2022). Health sector strategic plan III (HSSP III) 2023–2030: reforming for universal health coverage. https://dms.hiv.health.gov.mw/link/d9nbvzwn.

[23] The World Bank The World Bank in Malawi. https://www.worldbank.org/en/country/malawi/overview#1.

[24] Hussein MK (2004). 5 - Decentralisation and Development: The Malawian Experience. AD.

[25] Hussein M (2012). Decentralisation and Management Reforms on the Death Bed? Obstacles Facing Malawi’s District Councils. *BAFR*.

[26] (1995). The Constitution of the Republic of Malawi Act.

[27] Government of Malawi (1998). The Local Government Act, No.42 of 1998.

[28] Decentralization Secretariat (1998). Malawi decentralization policy.

[29] OECD/UCLG (2022). 2022 country profiles of the World Observatory on Subnational Government Finance and Investment: Malawi. https://www.sng-wofi.org/country_profiles/malawi.html.

[30] Government of Malawi (2017). Malawi health sector strategy plan III reforming for universal health coverage (2023-2030).

[31] Government of the Republic of Malawi (2023). National community health framework 2023–2030. https://www.hcwpolicylab.org/wp-content/uploads/2024/07/Malawi-National-Community-Health-Framework-2023-2030.pdf.

[32] Munthali AC, Kalumba J, Mkombe D (2021). Political economy analysis of health management in eastern and southern Africa: Malawi country report.

[33] Chansa C, Pattnaik A (2018). Expanding health care provision in a low-income country: the experience of Malawi.

[34] National AIDS Commission (Malawi) (2025). Malawi HIV factsheet 2025. https://www.aidsmalawi.org.mw/wp-content/uploads/2025/08/Malawi-HIV-Factsheet-2025.pdf.

[35] Connolly E, Zhuwao F, Rosenthal A Developmental and operationalisation influences of Malawi’s health sector strategic plan iii 2023-2030: a qualitative study of the context, processes, content and actors. Health Policy.

[36] Ministry of Health, Malawi (2018). Malawi population-based HIV impact assessment (MPHIA) 2015–2016: final report. https://phia.icap.columbia.edu/wp-content/uploads/2019/08/MPHIA-Final-Report_web.pdf.

[37] Exemplars in Global Health (2025). Empowering local people on the front lines: Dr. Gift Kawalazira, District Health Director Blantyre, Malawi on why a successful HIV prevention strategy is like a Jigsaw puzzle. https://www.exemplars.health/stories/empowering-local-people-on-the-front-lines-dr-gift-kawalazira-district-health-director-blantyre-malawi-on-why-a-successful-hiv-prevention-strategy-is-like-a-jigsaw-puzzle.

[38] Carter AM, Mablekisi C, Kawalazira G, Katz R, Boyce M (2021). Inoculating cities: case studies of urban pandemic preparedness.

[39] Kawalazira G, Kamgwira Y, Allinder SM (2025). A health systems approach to more effective decentralised HIV prevention: development of Malawi’s Blantyre Prevention Strategy. BMJ Glob Health.

[40] African Population & Health Research Center (APHRC) (2025). Evaluation of the Blantyre prevention strategy: program design, implementation and impact report. https://aphrc.org/publication/evaluation-of-the-blantyre-prevention-strategy-program-design-implementation-and-impact-report/.

[41] Allinder SM, Moses E, Enock M (2025). Using quality improvement to close HIV prevention gaps and strengthen district health systems: Blantyre, Malawi’s approach and early implementation. *Front Reprod Health*.

[42] Saunders C, Carter DJ (2017). Is health systems integration being advanced through Local Health District planning?. Aust Health Rev.

[43] Liwanag HJ, Wyss K (2019). Optimising decentralisation for the health sector by exploring the synergy of decision space, capacity and accountability: insights from the Philippines. Health Res Policy Syst.

[44] Liwanag HJ, Wyss K (2018). What conditions enable decentralization to improve the health system? Qualitative analysis of perspectives on decision space after 25 years of devolution in the Philippines. PLoS One.

[45] George AS, Mehra V, Scott K (2015). Community Participation in Health Systems Research: A Systematic Review Assessing the State of Research, the Nature of Interventions Involved and the Features of Engagement with Communities. PLoS One.

[46] Segall M (2003). District health systems in a neoliberal world: a review of five key policy areas. Int J Health Plann Manage.

[47] Buse C (2013). Intersectoral action for health equity. J Eval Clin Pract.

[48] Amri M, Chatur A, O’Campo P (2022). Intersectoral and multisectoral approaches to health policy: an umbrella review protocol. Health Res Policy Syst.

[49] Bosire EN, Mwapasa V, Mendenhall E (2025). Engaging Councillors to Address Structural and Social Drivers of HIV Infections in Blantyre City: A Formative Study. Int J Health Policy Manag.

[50] Blantyre Prevention Strategy Engaging political leaders. https://www.blantyrepreventionstrategy.com/engaging-political-leaders.

[51] Institute of Medicine (2014). Investing in global health systems: sustaining gains, transforming lives.

[52] United Nations, Office of the Special Envoy for Haiti (2012). Can more aid stay in Haiti and other fragile settings? How local investment can strengthen governments and economies. https://lessonsfromhaiti.org/download/Report_Center/osereport2012.pdf.

[53] Japan Center for International Exchange (2008). Task force on global action for health system strengthening, G8 Hokkaido Toyako summit follow-up global action for health system strengthening policy recommendations to the G8. https://jcie.org/researchpdfs/takemi/full.pdf.

[54] National Academy of Medicine (2016). The neglected dimension of global security: a framework to counter infectious disease crises.

[55] Azizatunnisa’ L, Cintyamena U, Mahendradhata Y (2021). Ensuring sustainability of polio immunization in health system transition: lessons from the polio eradication initiative in Indonesia. BMC Public Health.

[56] Kawonga M, Blaauw D, Fonn S (2016). The influence of health system organizational structure and culture on integration of health services: the example of HIV service monitoring in South Africa. Health Policy Plan.

[57] Kawonga M, Fonn S, Blaauw D (2013). Administrative integration of vertical HIV monitoring and evaluation into health systems: a case study from South Africa. Glob Health Action.

[58] Matanje B, Masha RL, Rwibasira G (2025). The global HIV response at a crossroads: protecting gains and advancing sustainability amid funding disruptions. Lancet HIV.

[59] Topp SM, Otiso L, Kawalazira G (2026). Advancing functional and systemic integration of HIV prevention into public health systems. Lancet Glob Health.

[60] The Global Fund (2022). Guidance note: sustainability, transition and co-financing. https://www.theglobalfund.org/media/5648/core_sustainabilityandtransition_guidancenote_en.pdf.

